# HBV DNA polymerase upregulates the transcription of PD-L1 and suppresses T cell activity in hepatocellular carcinoma

**DOI:** 10.1186/s12967-024-05069-y

**Published:** 2024-03-12

**Authors:** Yan Jia, Jianing Zhao, Chunqing Wang, Jing Meng, Liqing Zhao, Hongwei Yang, Xiaoqing Zhao

**Affiliations:** 1 Department of Laboratory Medicine, Tianjin Hospital, Tianjin, 300211 China; 2 Department of Pathology, The Fourth Hospital of Hebei Medical University, Shijiazhuang, Hebei 050011 China; 3 Department of Clinical Laboratory Medicine, The First Affiliated Hospital of Shandong First Medical University & Shandong Provincial Qianfoshan Hospital, Shandong Medicine and Health Key Laboratory of Laboratory Medicine, Jinan, 250014 China; 4 Department of Clinical Laboratory, The Second Hospital, Cheeloo College of Medicine, Shandong University, Jinan, 250033 China; 5 Department of Pediatrics, Zaozhuang Municipal Hospital, Zaozhuang, 277100 China

**Keywords:** Hepatocellular carcinoma, T cell dysfunction, HBV DNA polymerase, PD-L1, PARP1

## Abstract

**Background:**

In HBV-associated HCC, T cells often exhibit a state of functional exhaustion, which prevents the immune response from rejecting the tumor and allows HCC to progress. Moreover, polymerase-specific T cells exhibit more severe T-cell exhaustion compared to core-specific T cells. However, whether HBV DNA polymerase drives HBV-specific CD8^+^ T cell exhaustion in HBV-related HCC remains unclear.

**Methods:**

We constructed a Huh7 cell line stably expressing HA-HBV-DNA-Pol and applied co-culture systems to clarify its effect on immune cell function. We also examined how HBV-DNA-Pol modulated PD-L1 expression in HCC cells. In addition, HBV-DNA-Pol transgenic mice were used to elucidate the underlying mechanism of HBV-DNA-Pol/PD-L1 axis-induced T cell exhaustion.

**Results:**

Biochemical analysis showed that Huh7 cells overexpressing HBV-DNA-Pol inhibited the proliferation, activation, and cytokine secretion of Jurkat cells and that this effect was dependent on their direct contact. A similar inhibitory effect was observed in an HCC mouse model. PD-L1 was brought to our attention during screening. Our results showed that the overexpression of HBV-DNA-Pol upregulated PD-L1 mRNA and protein expression. PD-L1 antibody blockade reversed the inhibitory effect of Huh7 cells overexpressing HBV-DNA-Pol on Jurkat cells. Mechanistically, HBV-DNA-Pol interacts with PARP1, thereby inhibiting the nuclear translocation of PARP1 and further upregulating PD-L1 expression.

**Conclusions:**

Our findings suggest that HBV-DNA-Pol can act as a regulator of PD-L1 in HCC, thereby directing anti-cancer immune evasion, which further provides a new idea for the clinical treatment of liver cancer.

**Supplementary Information:**

The online version contains supplementary material available at 10.1186/s12967-024-05069-y.

## Background

Hepatocellular carcinoma (HCC) is the sixth most common cancer worldwide and the fourth leading cause of cancer-related death [1]. Chronic hepatitis B virus (HBV) infection is a high-risk factor for hepatocellular carcinoma (HCC), accounting for 50–80% of HCC cases worldwide [2]. During chronic HBV infection in humans, adaptive immunity changes from immune tolerance to progressive immune activation, inactivation, reactivation, and exhaustion, all of which may be immune pathogenic factors in the development of HCC [3]. CD8^+^ T cells are a major immune cell type that exert antivirus/antitumor activity. However, in HBV-related HCC, CD8^+^ T cells display a dysfunctional state known as exhausted T-cell that render the immune response unable to eliminate the virus or reject the tumors [4]. However, what induces T-cell exhaustion in HBV-related HCC and whether chronic HBV infection drives T-cell dysfunction has not been well characterized.

The HBV genome encodes four proteins with overlapping open reading frames: surface protein (S), core protein (C), polymerase, and a multifunctional nonstructural protein called X (HBx) [5]. HBV DNA polymerase is the only enzymatically active protein that participates in several steps of the viral replication cycle. HBV DNA polymerase can be divided into four sections: terminal protein (TP) domain, reverse transcriptase (RT) domain, RNase H-like (RH) domain, and spacer domain [6]. Previous studies of HBV DNA polymerase have focused on viral replication. Recent reports have shown that HBV DNA polymerase can also exhibit immunomodulatory activity by binding to a range of host factors [7]. Moreover, polymerase-specific CD8^+^ T cells exhibited severer T cell exhaustion and lower expansion capacity in chronic HBV-infected individuals with lower viral loads compared to core-specific CD8^+^ T cells [8]. However, little is known about whether and how HBV DNA polymerase drives HBV-specific CD8^+^ T cell exhaustion in HBV-related HCC.

Surveillance by the immune system makes it difficult for tumor cells to survive, so in order to preserve themselves, they continuously express some immune checkpoint molecules at a high level on the cell surface, thus inhibiting the function of T-cells, and in this way avoiding attacks from immune cells, and consequently immune escape occurs. Programmed death ligand-1 (PD-L1) is one of these immune checkpoints, which can inhibit the function of immune cells by binding to programmed death-1 (PD-1) expressed on it, thereby allowing tumor cells to escape [9]. Over the past decade, therapies that block PD-L1 binding to PD-1 have been quite effective in many late-stage cancers, as well as HCC [10]. Several studies have shown that PD-L1 overexpression, tumors continue to grow uncontrollably, leading to a worse prognosis for cancer patients [11, 12]. Moreover, high AFP levels and mal-differentiated tumors were associated with PD-L1 overexpression [13]. Tumor-infiltrating effector CD8^+^ T cells also upregulate PD-1 and PD-L1 expression, which is also associated with disease development and relapse [14]. In tumor cells of HBV-associated HCC, PD-L1 is similarly elevated, but the mechanism by which HBV induces elevated PD-L1 expression is still not well understood, nor is it known whether HBV polymerase is involved.

Poly(adenosine diphosphate ribose) polymerase 1 (PARP1) belongs to the ADP-ribosyltransferase (PARP) family, which consists of three functional domains: the DNA binding domain (DBD), the auto-modification domain (AMD) and the CD domain, and it is a multifunctional protein present in most eukaryotic cells [15]. PARP1 has been reported to be involved in a variety of biological processes, such as chromatin strengthening, stress signaling, cell survival/death, inflammation, drug resistance, and differentiation [16, 17]. PARP1 also functions as a sensor of oxidative stress and PARP1 inhibitors have been shown to enhance the sensitivity of cancer cells to chemotherapy [18]. In addition, PARP1 regulates STAT3 [19] negatively, one of the most important transcription factors of PD-L1. However, whether PARP1 participates in the exhaustion of HBV-specific CD8^+^ T cells in HBV-related HCC remains unclear.

The current study investigates the role of HBV DNA polymerase in antitumor immunity. Here, we verified that HBV-DNA-Pol significantly inhibits the proliferation, activation, and cytokine secretion of Jurkat cells. We further demonstrated that HBV-DNA-Pol increased PD-L1 expression by interfering with the nuclear translocation of PARP1, a negative regulator of STAT3-mediated PD-L1 transcription in various cancers, through interaction with PARP1. In addition, the PD-L1 antibody blockade attenuated the suppression of immune cell function by HBV-DNA-Pol. Supporting this regulatory mechanism, our experiments in a transgenic HBV-DNA-Pol mouse model showed that HBV-DNA-Pol impairs the function of CD4^+^ and CD8^+^ T cells. In conclusion, our study revealed a regulatory role for HBV-DNA-Pol in PD-L1-mediated immune escape in HCC, suggesting that HBV DNA polymerase is a potential target for HCC therapy.

## Methods

### Cell culture and transfection

Jurkat, Huh7, HepG2, Hep3B, SMMC-7721 and HepG2.2.15 cells were obtained from the American Type Culture Collection. Huh7, HepG2, Hep3B, SMMC-7721 and HepG2.2.15 cells were maintained in Dulbecco’s Modified Eagle’s medium (Gibco, Grand Island, NY, USA). Jurkat cells were maintained in RPMI medium 1640 basic (Gibco). All media were supplemented with 10% fetal bovine serum (Gibco) and penicillin–streptomycin. Cells were cultured at 37 °C in a humidified incubator with 5% CO_2_. Cell transfection was performed using Lipofectamine 2000 (Invitrogen, CA, USA), following the manufacturer’s protocol.

Huh7 cell lines stably expressing HA-HBV-DNA-Pol and Control cell lines were screened with puromycin (2 μg/ml) for 7 days, beginning 48 h after lentivirus infection, and validated by western blotting.

### Cell co-culture and IFN-γ/TNF-α ELISA assay

HBV-DNA-Pol^+^ Huh7 cells or control cells were co-cultured with Jurkat cells at a ratio of 3:1 in complete RPMI medium 1640 basic (Gibco) supplemented with 10% fetal serum (Gibco) and penicillin–streptomycin for 24 h in 24-well plates with or without a well at 37 °C in a humidified incubator with 5% CO_2_. Here, “with well” means that the two cells are cultured in a non-contact manner, i.e., indirectly, and “without well” means that the two cells are mixed together and cultured in a contact manner, i.e., directly. The “well” is an experimental tool that allows the spatial separation of cells. Then Jurkat cells were sorted out because of their suspension properties and stimulated by adding 20 μg/ml Concanamycin A (Con A) for 24 or 48 h for subsequent experiments. The supernatants were collected after 24 h and examined using a human or mouse IFN-γ/TNF-α ELISA kit (Solarbio, Beijing, China) according to the manufacturer’s instructions, and the results were analyzed using ELISACalc V0.1 software (Quant, Biotek, Germany).

### Plasmids construction

To generate cell lines stably expressing HA-HBV-DNA-Pol, full-length HA-HBV-DNA-Pol was amplified by PCR using the primers 5′-ATT TCC GGT GAA TTC ATG TAC CCA TAC GAC GTC CCA GAC TAC GCT CCC CTA TCC TAT CAA-3′ and 5′-ATC CGC GGC CGC TCT AGA CGG TGG TCT CCA TGC GAC-3′ and cloned into the EcoRI and XbaI restriction sites of pLVX-IRES-puro vector (LMAI Bio, shanghai, China). To obtain HA-tagged HBV-DNA-Pol, full-length open reading frames (ORFs) were cloned into the KpnI and NotI sites of a modified pcDNA3 plasmid (Invitrogen) containing an N-terminal 3 × HA-tag by PCR using primers 5′-GCC GGT ACC ATG CCC CTA TCC TAT CAA CA-3′ and 5′-ATA AGA ATG CGG CCG CCG GTG GTC TCC ATG CGA C-3′ ORF truncations of HBV-DNA-Pol were constructed using PCR and cloned into the same sites of pcDNA3 with a 3 × HA-tag. Similarly, to obtain Flag-tagged PARP1, full-length open reading frames (ORFs) were cloned into the NotI and XbaI sites of pcDNA3 by PCR using the primers 5′-ATA AGA ATG CGG CCG CAT GGC GGA GTC TTC G-3′ and 5′-GCG TCT AGA CCA CAG GGA GGT CTT AAA-3′ ORF truncations of PARP1 were constructed using PCR and cloned into the same sites of pcDNA3 with a 3 × Flag-tag. To reduce PARP1 expression, 5′-GAT CCC GAC CTG ATC TGG AAC ATC AAC TCG AGT TGA TGT TCC AGA TCA GGT CGT TTT TGA-3′ and 5′-AGC TTC AAA AAC GAC CTG ATC TGG AAC ATC AAC TCG AGT TGA TGT TCC AGA TCA GGT CGG-3′ were synthesized and cloned into the BamHI and HindIII sites of pSilencer2.1-U6 neo (Ambion, 113P06).

### Antibodies and reagents

Anti-HA and anti-FLAG primary antibodies were purchased from Sigma-Aldrich (St. Louis, MO, USA). Anti-PARP1 primary antibody was purchased from Abcam (Cambridge, UK). Immunoaffinity-purified rabbit polyclonal antibodies against PD-L1, LMNB1, and GAPDH were obtained from Saier Biotech (Tianjin, China). Horseradish peroxidase-conjugated anti-mouse and anti-rabbit secondary antibodies were purchased from Zhongshan Golden Bridge Biotechnology (Beijing, China). The FITC/TRITC-conjugated secondary antibodies were purchased from Jackson ImmunoResearch Laboratories (West Grove, PA, USA). FITC-conjugated CD69 (human/mouse), APC-conjugated TNF-α (human/mouse), Brilliant Ultraviolet 615-conjugated IFN-γ (human/mouse), PE-Cy7-conjugated CD3 (mouse), PE-conjugated CD8 (mouse), APC-conjugated CD4 (mouse), and FITC-conjugated CD4 (mouse) were purchased from eBioscience (San Diego, CA, USA). Anti-PD-L1 (PE-conjugated CD274, human) was purchased from BD Biosciences (NJ, CA, USA).

Con A was obtained from Solarbio (Beijing, China), and actinomycin D (Act D), cycloheximide (CHX), and puromycin were purchased from Sigma-Aldrich. CellTrace CFSE was purchased from Invitrogen (Carlsbad, CA, USA). The compounds were dissolved in DMSO and used at the indicated concentrations.

### MTT assay

The MTT staining method, as described by Mosmann [20], was used with some modifications. Jurkat cells were seeded into 96-well plates at a density of 10^5^ cells/well. After a 24- or 48-h incubation period, 10 μl of 5 mg/ml MTT solution (Sigma) was added to each well and the plate was further incubated at 37 °C for 4 h. Thereafter, the medium was aspirated, MTT was dissolved in 100 µL DMSO (Sigma, MO, USA), and absorbance was read at 570 nm using a Quant Universal Microplate Spectrophotometer (Quant, Biotek, Germany).

### Flow cytometry

Single-cell suspensions were stained with the indicated fluorescence-conjugated primary antibodies for 30 min at 4 °C and then analyzed using high-end analytical flow cytometry (BD LSRFortessa). For intracellular staining, the cells were permeabilized prior to incubation with antibodies. The results were analyzed using FlowJo V10 software (Tree Star Inc., Ashland, OR, USA).

### Reverse transcription (RT) and quantitative PCR (qPCR) analysis

Total RNA was extracted using RNAiso Plus reagent (Takara, Dalian, China). cDNA was generated using PrimeScript RT Master Mix (Takara), according to the manufacturer's protocol. The cDNA was quantified by real-time PCR using a real-time fluorescent quantitative PCR instrument (LightCycler 480, Roche) with SYBR Green PCR Master Mix (Takara). Relative quantification was expressed as 2^−ΔCt^, where Ct is the difference between the main Ct value of triplicates of the sample and that of the GAPDH mRNA control. The quantitative PCR primers used are listed in Table 1.

**Table 1:** The primers for real-time PCR

Genes	Forward Primers (5′-3′)	Reverse Primers (5′-3′)
GAPDH	CAAAATGGTGAAGGTCGGTGT	TGATGTTAGTGGGGTCTCGCT
HBV-P	CTCTCTTTACGCGGACTCCC	TCTTCTAGGGGACCTGCCTC
PD-L1	GCCGAAGTCATCTGGACAAG	TCTCAGTGTGCTGGTCACAT
CD73	CCAGTACCAGGGCACTATCTG	TGGCTCGATCAGTCCTTCCA
CD47	GGCAATGACGAAGGAGGTTA	ATCCGGTGGTATGGATGAGA
CD146	CCAAGGCAACCTCAGCCATGTC	GCAGGGTCTGACACCTCAGCTC
CD155	TATCTGGCTCCGAGTGCTTGCC	ACGACGGCTGCAAAAGTGGCG
ICAM1	CCCTTCCCCCCAAAACTG	GTCATTGTGAACACTGGCAGAAA
CD74	CCAGCGAGGAGCAGAGTCAC	TTATCTCCAACAATGAGCAACT
IDO	AGCGTCTTTCAGTGCTTTG	GGATTTGACTCTAATGAGCACA
B7H3	CACAGGAAGATGCTGCGTCG	CAATGAGACAGACAGACAGC
CD69	CATAGCTCTCATTGCCTTATCAGT	CCTCTCTACCTGCGTATCGTTT

### Western blot analysis

Cells were harvested, and the proteins were extracted with RIPA lysis buffer (1 mM MgCl_2_, 10 mM Tris–HCl pH 7.4, 0.1% SDS, 1% NP-40). Protein expression was analyzed by western blotting as described previously [21].

### Immunofluorescence microscopy

For immunostaining, Huh7 cells were fixed in 4% paraformaldehyde at room temperature for 20 min. The fixed cells were then rinsed with ice-cold 1 × PBS and permeabilized with 200 μl PBST solution (0.1% Triton X-100 in PBS) at 4 °C for 10 min. The cells were rinsed with PBS for 5 min and blocked for nonspecific interactions with 4% BSA in PBS solution at 37 °C for 30 min. The cells were incubated with the corresponding antibodies overnight at 4 °C. After washing with 1 × PBS, the slides were incubated with TRITC/FITC-conjugated secondary antibody for 2 h at 4 °C. The nuclei were counterstained with 0.05 g/ml DAPI in PBS for 1 min at RT. The slides were imaged using NIS software with an ECLIPSE TS2 confocal microscope (Zeiss, Thornwood, NY).

### Mass spectrometry (MS)

To identify proteins associated with HBV DNA polymerase, affinity purification was performed, followed by MS analysis. Total protein extracts from Huh7 cells expressing HA-HBV-P were prepared and incubated overnight at 4 °C with protein A/G beads encapsulated with anti-HA antibodies. The beads were collected by centrifugation at 400*g* for 5 min and washed four times with the co-IP buffer. The beads were then boiled in SDS loading buffer and quickly added to SDS gels. Gel sections containing the purified proteins were isolated for MS analysis.

### Co-immunoprecipitation

For co-immunoprecipitation, the cells were lysed in lysis buffer (Tris–HCl 50 mM, pH 8.0; NaCl 150 mM, EDTA 5 mM, 0.5% NP-40). Lysates were incubated with antibodies on a rotating wheel overnight at 4 °C and then pulled down using protein A/G agarose beads (Bioworld Technology, Louis, USA) at 4 °C for 6 h. Beads were collected by centrifugation, washed, boiled in 2 × SDS-PAGE sample buffer, and analyzed by western blotting.

### Preparation of cytoplasmic and nuclear fractions

Subfractionation of transfected cells into nuclear and cytoplasmic extracts was performed as previously described by Goodkin et al. [22]. Briefly, 48 h after transfection, 2 × 10^6^ cells were collected by trypsin/EDTA treatment and resuspended in 1 × PBS. After resuspension in 150 μl of PBS containing 0.4% NP-40, the cell pellet was lysed by gentle inversion, followed by centrifugation at 1000×*g* for 3 min. Supernatants (cytoplasmic extracts) were removed and placed in fresh tubes. The nuclear pellet was washed once with 100 µL PBS containing 0.1% NP-40 and resuspended in 100 µL RIPA buffer (nuclear extract).

### EdU incorporation

The BeyoClick EdU Cell Proliferation Kit with Alexa Fluor 488 was purchased from Beyotime (Shanghai, China). Cells were subjected to Edu (5-ethynyl-2′-deoxyuridine) incorporation experiments according to the manufacturer's protocol, followed by flow assays to detect cell proliferation.

### Immunohistochemistry

All slides were microwave-heated with sodium citrate buffer for 15 min after de-paraffinization for antigen repair, and then incubated with 3% hydrogen peroxide for 10 min, followed by 30 min in blocking buffer (5% goat serum in PBS). The slides were then probed with a 1:50 dilution of anti-PD-L1, treated with a biotinylated secondary antibody and horseradish peroxidase-conjugated avidin, and visualized with 3,3-diaminobenzidine according to the manufacturer's protocol. The digital images were quantified using Image-Pro Plus 6.0 software (version 6.0, Media Cybernetics).

### Protein half-life detection

Cells were treated with cycloheximide (CHX, Selleck, USA) to measure the half-lives of the proteins. Briefly, the cells were cultured with 100 μg/ml CHX for 0, 2, 4 and 6 h in logarithmic growth phase and then collected for Western blot analysis.

### RNA half-life measurement

Cells were mixed with actinomycin D (5 µg/ml, AAT Bioquest) to block the synthesis of new RNA in the cells. Next, qRT-PCR was performed at the indicated time points (0 h, 1 h, 2 h, 3 h,4 h) for stability analysis of PD-L1 mRNA.

### HCC mouse model

Mice expressing Flag-HBV-DNA-Pol, specifically in the liver tissue of mice with a C57BL/6J background, were maintained and treated in accordance with the ethical guidelines approved by the Institutional Animal Care and Use Committee (IACUC) at MD Anderson. Regarding the construction of this mouse, we designed the sequence ourselves, and then the construction was performed using Cyagen. We inserted the mouse albumin promoter (538 bp) between NdeI and HindIII of pcDNA3, which replaced the original CMV promoter, followed by the insertion of the 3 × Flag sequence between HindIII and KpnI, the HBV-DNA polymerase gene (2499 bp) between KpnI and NotI, and the BGH ployA sequence between NotI and BbsI. The total sequence length was 3403 bp. For the HCC mouse model, HBV-DNA-Pol transgenic male mice and sibling control mice were injected intraperitoneally with diethylnitrosamine (DEN) (20 mg/kg) on postnatal day 14, and 20% CCl_4_ (5 ml/kg) was injected intraperitoneally twice a week for 14 weeks starting from the 3rd week. Mice were sacrificed at 20 weeks to observe tumorigenesis and successful mice were selected for subsequent experiments.

### Statistics

Statistical significance was determined using two-tailed homoscedastic Student’s t-test. For all data analyzed, values were expressed as the mean ± standard deviation (S.D.), and statistical significance was set at P < 0.05. The data generated are representative of at least three separate experiments.

## Results

### pLVX-HA-HBV-P cells inhibits the function of Jurkat cells

**Fig. 1:**
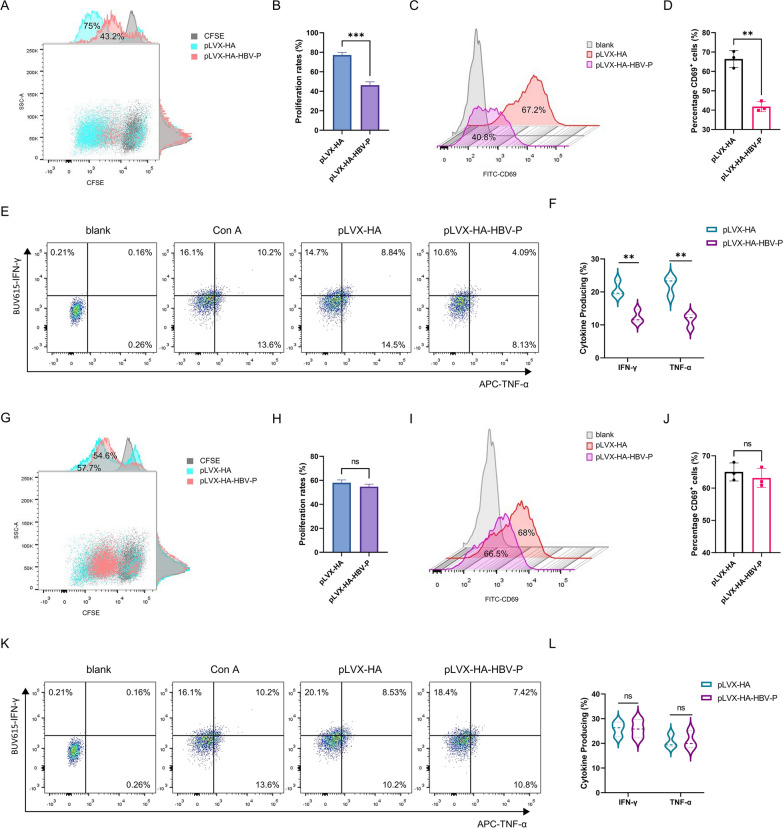
HBV-DNA-Pol^+^ Huh7 cells inhibited the function of Jurkat cells. **A**, **B** HBV DNA polymerase inhibites the proliferation of Jurkat cells after direct co-culture. **A** Jurkat cells were directly co-cultured with HBV-DNA-Pol^+^ Huh7 cells or control cells for 24 h, then isolated and stained with CFSE, flow cytometry was used to measure proliferation rates after 48 h of Con A stimulation. **B** Results are plotted after quantification. **C**, **D** HBV DNA polymerase inhibits Jurkat cell activation after direct co-culture. **C** Jurkat cells were directly co-cultured with HBV-DNA-Pol^+^ Huh7 cells or control cells for 24 h, then isolated and CD69 expression was detected by flow cytometry after 24 h of Con A stimulation. **D** Quantification results are shown. **E**, **F** HBV DNA polymerase inhibits cytokine secretion in Jurkat cells after direct coculture. **E** Jurkat cells were directly co-cultured with HBV-DNA-Pol^+^ Huh7 cells or control cells for 24 h and then isolated to measure IFN-γ and TNF-α production by flow cytometry 24 h after Con A stimulation. **F** IFN-γ and TNF-α production was summarized. **G**, **H** HBV DNA polymerase does not impair the proliferation of Jurkat cells after indirect co-culture. **G** Jurkat cells were indirectly co-cultured with HBV-DNA-Pol^+^ Huh7 cells or control cells for 24 h, isolated and stained with CFSE, and proliferation rates were measured by flow cytometry after 48 h of Con A stimulation. **H** Results are plotted after quantification. **I**, **J** HBV DNA polymerase does not impair the activation of Jurkat cells after indirect co-culture. **I** Jurkat cells were indirectly co-cultured with HBV-DNA-Pol^+^ Huh7 cells or control cells for 24 h, then isolated and CD69 expression was determined by flow cytometry after 24 h of Con A stimulation. **J** Quantification results are shown. **K**, **L** HBV DNA polymerase does not impair cytokine secretion in Jurkat cells after indirect coculture. **K** Jurkat cells were indirectly co-cultured with HBV-DNA-Pol^+^ Huh7 cells or control cells for 24 h and then isolated to measure IFN-γ and TNF-α production by flow cytometry 24 h after Con A stimulation. **L** IFN-γ and TNF-α production was summarized. Three independent experiments were performed for all statistical comparisons (mean ± S.D., n = 3, Student’s t-test). **P < 0.01, ***P < 0.001, ns, no significant

To explore the biological role of HBV-DNA-Pol in T cell exhaustion, we constructed Huh7 cell lines stably overexpressing HA-HBV-DNA-Pol (pLVX-HA-HBV-P) and control cell lines (pLVX-HA) (Additional file 1: Fig. S1A), and co-cultured them directly or indirectly with Jurkat cells. After 48 h, Jurkat cells were isolated and stimulated with ConA. Jurkat cells were then examined for proliferation, activation, and cytokine secretion at specific times. The results showed that when co-cultured directly with Jurkat cells, pLVX-HA-HBV-P cells inhibited the proliferation (Fig. 1A, B and Additional file 1: Fig. S1B), activation (Fig. 1C, D and Additional file 1: Fig. S1C), and cytokine secretion (Fig. 1E, F and Additional file 1: Fig. S1D, E) of Jurkat cells. However, when co-cultured indirectly with Jurkat cells, pLVX-HA-HBV-P cells did not affect the proliferation (Fig. 1G, H and Additional file 1: Fig. S1F), activation (Fig. 1I, J and Additional file 1: Fig. S1G), or cytokine secretion (Fig. 1K, L and Additional file 1: Fig. S1H, I) of Jurkat cells. In conclusion, these results suggest that pLVX-HA-HBV-P cells inhibit the functions of Jurkat cells and that this inhibition is achieved through immune checkpoint molecule receptor ligand binding rather than soluble cytokine secretion.

### HBV DNA polymerase upregulates PD-L1 expression at the transcriptional level

**Fig. 2:**
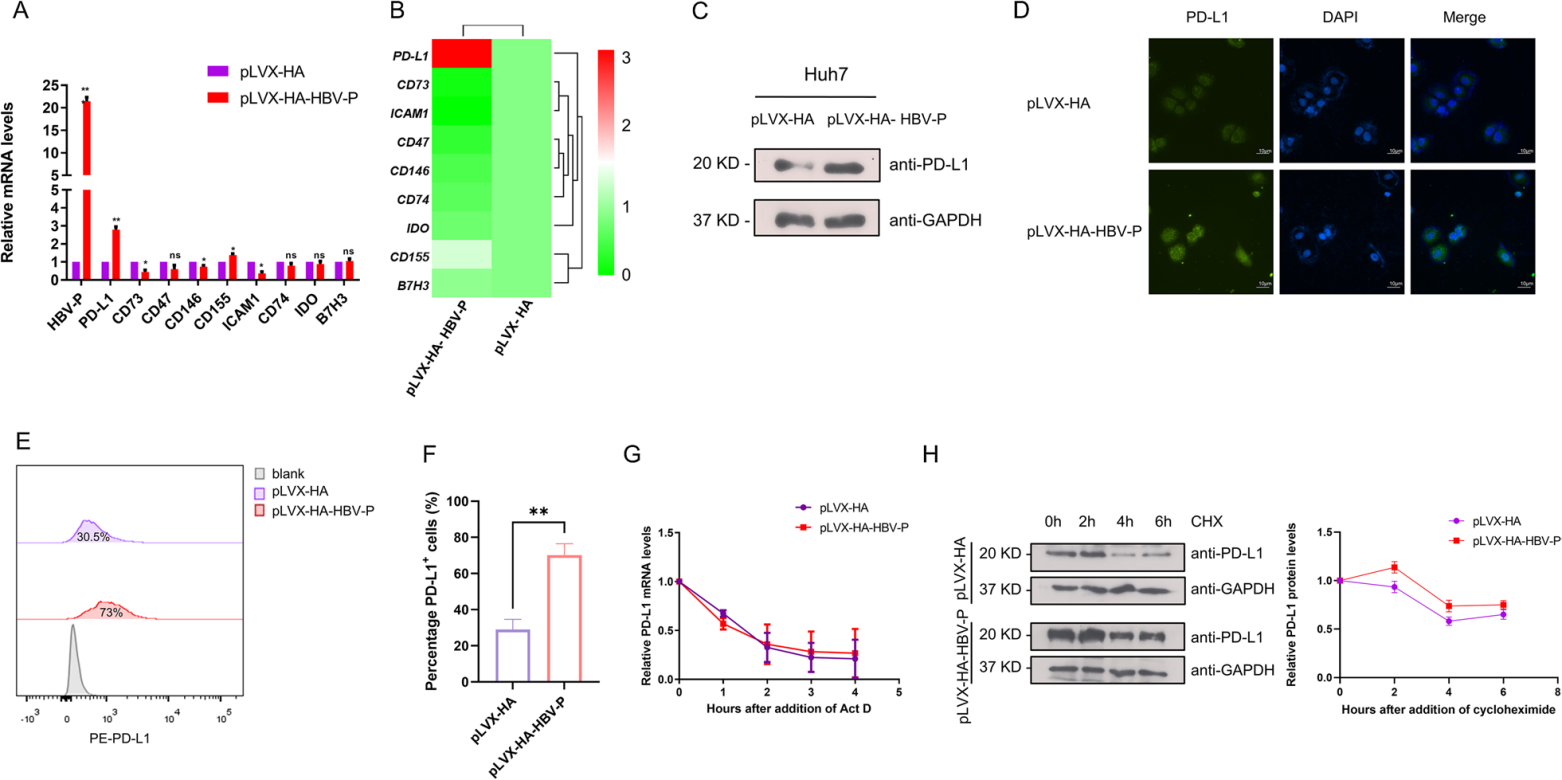
HBV-DNA-Pol upregulates PD-L1 expression at the transcriptional level. **A**, **B** PD-L1 was identified as a differential immune checkpoint molecule. **A** RNA extracts from HBV-DNA-Pol^+^ Huh7 cells or control cells were harvested and analyzed using qRT-PCR to determine the levels of relevant immune checkpoint molecules. GAPDH mRNAs was used as a control. Three independent experiments were performed for all statistical comparisons (mean ± S.D., n = 3, Student’s t-test). *P < 0.05, **P < 0.01, ***P < 0.001, ns, no significant. **B** Heat map based on the RT-qPCR results. **C–E** HBV-DNA-Pol increases PD-L1 protein levels. **C** Extracts from HBV-DNA-Pol^+^ Huh7 cells or control cells were collected and analyzed by western blot analysis with anti-PD-L1 and anti-GAPDH antibodies. **D** HBV-DNA-Pol^+^ Huh7 cells or control cells were subjected to immunofluorescence staining with an antibody against PD-L1 and stained with DAPI to visualize the nuclei. Images were digitally merged. Scale bar, 10 μm. **E** Cell-surface PD-L1 was measured by flow cytometry in HBV-DNA-Pol^+^ Huh7 or control cells. The results are plotted after quantitation **(F)**. Three independent experiments were performed for all statistical comparisons (mean ± S.D., n = 3, Student’s t-test). ***P < 0.001. **G** HBV-DNA-Pol does not affect PD-L1 mRNA stability. HBV-DNA-Pol^+^ Huh7 cells or control cells were treated with 1 μg/ml Actinomycin D (ActD) for the indicated periods and then harvested for PD-L1 mRNA detection. **H** HBV-DNA-Pol does not affect the stability of PD-L1. HBV-DNA-Pol^+^ Huh7 cells or control cells were treated with 500 μg/ml cycloheximide (CHX) for the indicated periods and then harvested for immunoblotting with anti-PD-L1 and anti-GAPDH antibodies (left). The results are plotted after quantification (right)

Since pLVX-HA-HBV-P cells inhibit Jurkat cell function through receptor ligand binding, suggesting that HBV-DNA-Pol may affect the expression of immune checkpoint molecules in HCC cells, we therefore used qRT-PCR to detect the expression of immune checkpoint molecules in both pLVX-HA and pLVX-HA-HBV-P cell lines, including genes encoding PD-L1, CD73, CD47, CD146, CD155, ICAM1, CD74, IDO, and B7H3. It was found that the expression levels of PD-L1 and CD155 mRNA were elevated, while the expression levels of CD73, CD146, and ICAM1 mRNA were decreased in pLVX-HA-HBV-P cells compared to control cell lines (Fig. 2A and B).The expression of ICAM1 is also decreased in the results obtained from Hongxia et al. [23]. PD-L1, owing to its more pronounced elevation of mRNA and central role in immune evasion in many cancers, has attracted our attention. The expression of PD-L1 in different hepatocellular carcinoma cell lines was analyzed and we discovered that PD-L1 expression was highest in HepG2.2.15 cells, and PD-L1 expression further increased after overexpression of HBV-DNA-Pol in HepG2.2.15 cells, which also indicated the positive regulation of PD-L1 by HBV-DNA-Pol (Additional file 1: Fig. S2A, B).To further confirm the involvement of HBV-DNA-Pol in PD-L1 regulation, we examined the expression of PD-L1 protein by western blotting and immunofluorescence, and found that overexpression of HBV-DNA-Pol similarly upregulated the expression levels of PD-L1 protein (Fig. 2C and D). Flow cytometry also demonstrated that HBV-DNA-Pol overexpression upregulated the expression of PD-L1 on the surface of HCC cells (Fig. 2E and F). To clarify whether the regulation of PD-L1 by HBV-DNA-Pol is dose-dependent, we transfected different doses of HBV-DNA-Pol in Huh7 cells, and then detected the expression of PD-L1 by Western blotting. The results showed dose-dependent upregulation of PD-L1 expression by HBV-DNA-Pol. (Additional file 1: Fig. S2C). The present results suggest that the regulation of PD-L1 by HBV-DNA-Pol may occur at the transcriptional level (promoting transcription) or at the post-transcriptional level (maintaining mRNA stability). To determine the mechanism by which HBV-DNA-Pol increases PD-L1 abundance in HCC cells, we performed qRT-PCR in HCC cells to examine the effect of HBV-DNA-Pol on the half-life of PD-L1 mRNA. The data showed that HBV-DNA-Pol overexpression had little effect on the half-life of the PD-L1 mRNA (Fig. 2G). We then examined the effect of HBV-DNA-Pol on the stability of the PD-L1 protein. We used a cycloheximide (CHX, a protein synthesis inhibitor) chase assay to evaluate the protein stability of PD-L1. As shown in Fig. 2H, overexpression of HBV-DNA-Pol in Huh7 cells did not influence the half-life of the PD-L1 protein. Taken together, these data suggest that HBV-DNA-Pol increases PD-L1 expression in HCC cells by promoting its transcription.

### HBV DNA polymerase interacts with PARP1 in the cytoplasm

**Fig. 3:**
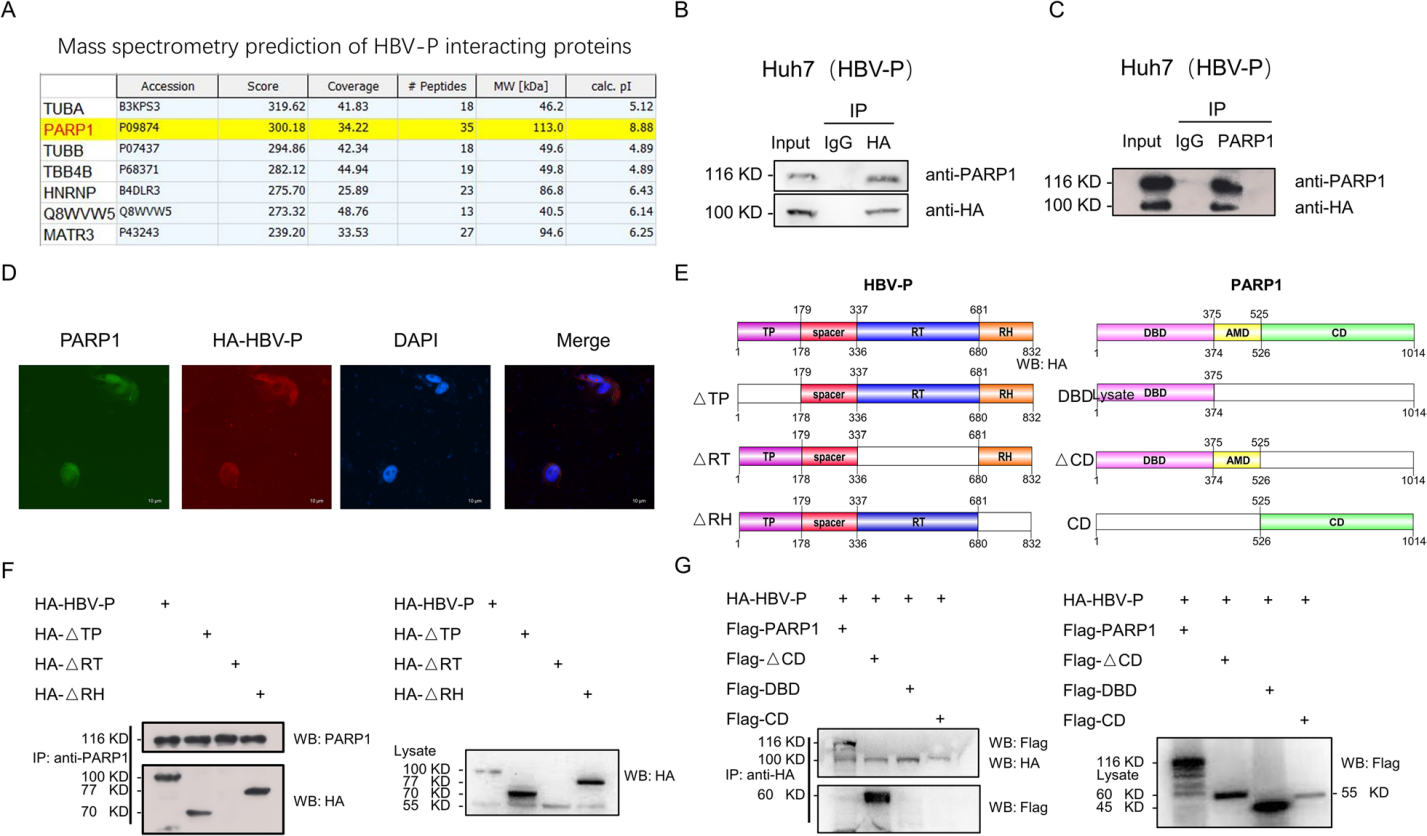
HBV-DNA-Pol interacts with PARP1. **A–C** HBV-DNA-Pol interacts with PARP1. **A** Mass spectrometry predicts a possible interaction of PARP1 with HBV-DNA-Pol. **B** Immunoprecipitation assays of HBV-DNA-Pol^+^ Huh7 cells were performed with anti-HA antibody and Western blotting with anti-PARP1 and anti-HA antibodies. **C** Immunoprecipitation assays of HBV-DNA-Pol^+^ Huh7 cells were performed with anti-PARP1 antibody and Western blotting with anti-HA and anti-PARP1 antibodies. **D** HBV-DNA-Pol co-localizes with PARP1 in the cytoplasm. Immunofluorescence assays of HBV-DNA-Pol^+^ Huh7 cells were performed with the indicated antibodies, then imaged by confocal microscopy. Scale bar, 10 μm. **E** Schematic diagram of full-length and truncated mutants of HBV-DNA-Pol (a) and PARP1 (b). **F** Mapping the PARP1-interacting site in HBV-DNA-Pol. Huh7 cells were transfected with the indicated plasmids. After 48 h, cell lysates were immunoprecipitated with anti-PARP1 antibody and then Western blotting with anti-HA and anti-PARP1 antibodies. **G** Mapping the HBV-DNA-Pol-interacting site in PARP1. HBV-DNA-Pol^+^ Huh7 cells were transfected with the indicated plasmids encoding Flag-tagged full-length PAPR1 or PARP1 deletion mutants. Immunoprecipitation assays were performed with anti-HA antibody and Western blotting with anti-PARP1 and anti-HA antibodies. All results are representative of three independent experiments

As there are no relevant studies on the direct involvement of HBV-DNA-Pol as a transcription factor in transcriptional regulation, we focused on its interacting proteins to further delineate the molecular mechanism of HBV-DNA-Pol-regulated PD-L1 transcription. HBV-DNA-Pol was immunoprecipitated in Huh7 cells and mass spectrometry was used to analyze its binding proteins. Intriguingly, PARP1 was among the candidate proteins that interacted with HBV-DNA-Pol (Fig. 3A), which has been shown to negatively regulate the expression of PD-L1 in previous studies [24, 25]. To verify the predicted results of mass spectrometry, we confirmed the interaction between HBV-DNA-Pol and endogenous PARP1 using co-IP in pLVX-HA-HBV-P HCC cells (Fig. 3B and C). Moreover, immunofluorescence (IF) showed that HBV-DNA-Pol and PARP1 co-localized in the cytoplasm (Fig. 3D). To identify which domain is involved in the interaction between HBV-DNA-Pol and PARP1, we constructed three HBV-DNA-Pol deletion mutants and three PARP1 truncation mutants and evaluated them by co-IP experiments (Fig. 3E). Co-IP assays in Huh7 cells indicated that deletion of the HBV-DNA-Pol-RT domain abolished its interaction with PARP1 (Fig. 3F). Additionally, we found that the AMD domain of PARP1 is involved in its interaction with HBV-DNA-Pol (Fig. 3G). These data indicate that PARP1 is an HBV-DNA-Pol-interacting protein that localizes to HBV-DNA-Pol in the cytoplasm of HCC cells.

### HBV DNA polymerase upregulates PD-L1 expression by repressing PARP1 nuclear translocation

**Fig. 4:**
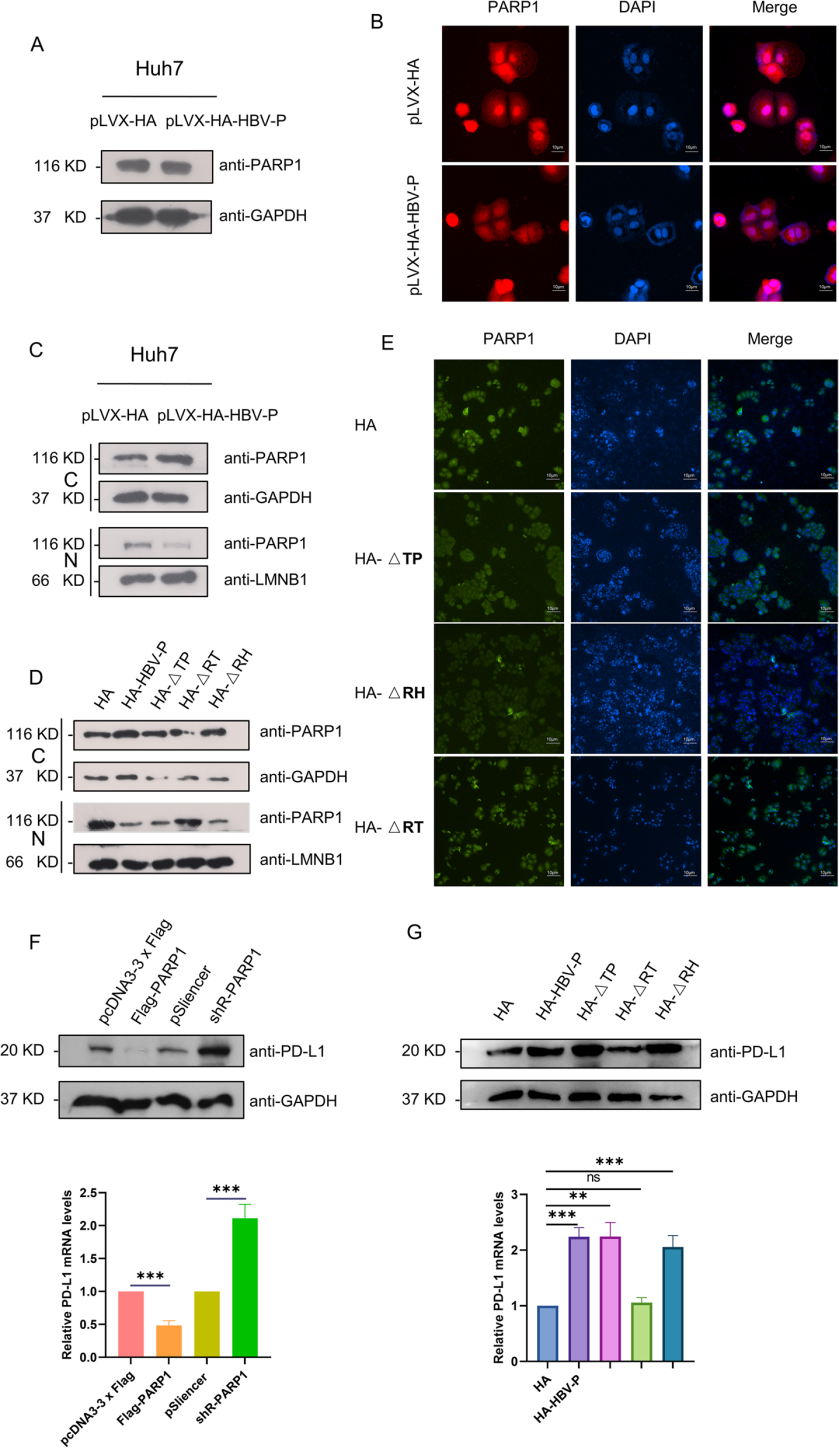
HBV-DNA-Pol upregulates PD-L1 expression via PARP1. **A** HBV-DNA-Pol does not impact PARP1 protein levels. Extracts from the HBV-DNA-Pol^+^ Huh7 cells or control cells were collected and subjected to Western blotting with anti-PARP1 and anti-GAPDH antibodies. **B**, **C** HBV-DNA-Pol inhibits nuclear translocation of PARP1. **B** HBV-DNA-Pol^+^ Huh7 cells or control cells were subjected to immunofluorescent staining with antibody against PARP1 and stained with DAPI to visualize the nuclei. The images were digitally merged. Scale bar, 10 μm. **C** Western blotting were used to detect PARP1 protein levels in nucleoplasm or cytoplasm from HBV-DNA-Pol^+^ Huh7 cells and control cells. **D**, **E** The effect of HBV-DNA-Pol on PARP1 is dependent on their interaction. **D** Huh7 cells were transfected with the indicated plasmids. Then nucleoplasmic or cytoplasmic extracts were harvested at 48 h post-transfection and analyzed by Western blotting with anti-PARP1, anti-GAPDH and anti-LMNB1 antibodies. **E** Huh7 cells were transfected with the indicated plasmids and then immunofluorescence assays were performed with anti-PARP1 antibody. The images were digitally merged. Scale bar, 10 μm. **F** PARP1 negatively regulates the expression of PD-L1. Huh7 cells were transfected with the indicated plasmids. Extracts were collected at 48 h post-transfection and PD-L1 expression was analyzed by Western blotting and qRT-PCR assays. **G** HBV-DNA-Pol upregulates PD-L1 expression via PARP1. Huh7 cells were transfected with the indicated plasmids. Extracts were collected at 48 h post-transfection and PD-L1 expression was analyzed by Western blotting and qRT-PCR assays

Next, we investigated whether the interaction between HBV-DNA-Pol and PARP1 affects PARP1 expression in HCC cells. Western blotting showed that PARP1 levels in pLVX-HA-HBV-P cells did not change compared to those in pLVX-HA cells (Fig. 4A). Interestingly, immunofluorescence microscopy showed that nuclear translocation of PARP1 appeared to be inhibited in HBV-DNA-Pol-overexpressing cells (Fig. 4B). Therefore, we speculated that binding to HBV-DNA-Pol may be important for the nucleocytoplasmic translocation of PARP1. This was further validated by subcellular fractionation of pLVX-HA and pLVX-HA-HBV-P cells, showing that HBV-DNA-Pol attenuated PARP1 trafficking from the cytoplasm to the nucleus (Fig. 4C). Notably, when the RT domain interacting with PARP1 in HBV-DNA-Pol was deleted, the inhibition of PARP1 nuclear translocation was alleviated (Fig. 4D, E). Previous studies have shown that PARP1 can negatively regulate PD-L1 expression through modulation of STAT3 after its entry into the nucleus [23, 24]. Based on our current findings and those of previous studies, we postulated that HBV-DNA-Pol-mediated upregulation of PD-L1 expression is dependent on PARP1 in HCC. Therefore, we first verified this regulatory role of PARP1 on PD-L1, transfection of Huh7 with PARP1 shRNA subsequently resulted in increased mRNA and protein levels of PD-L1, while PARP1 overexpression had the opposite effect (Fig. 4F), which is consistent with previous reports. Next, we performed rescue experiments by deleting the RT domain interacting with PARP1 in HBV-DNA-Pol and found that deletion of the RT domain alleviated the upregulation of PD-L1 caused by HBV-DNA-Pol (Fig. 4G). We also evaluated whether PARP1 overexpression could similarly exert this alleviative effect, and the results showed that the upregulation of PD-L1 by HBV-DNA-Pol was similarly alleviated when PARP1 was overexpressed (Additional file 1: Fig. S3A). Given that the regulation of PD-L1 by PARP1 is dependent on STAT3, we assessed the role of STAT3 in this context. As shown in Additional file 1: Fig. S3B, when knocking down STAT3, HBV-DNA-Pol no longer upregulated PD-L1 expression, which confirmed the previous findings. These results imply that HBV-DNA-Pol upregulates PD-L1 expression by inhibiting nuclear translocation of PARP1.

### PD-L1 blockade reverses the suppression of immune cell function by pLVX-HA-HBV-P cells

**Fig. 5:**
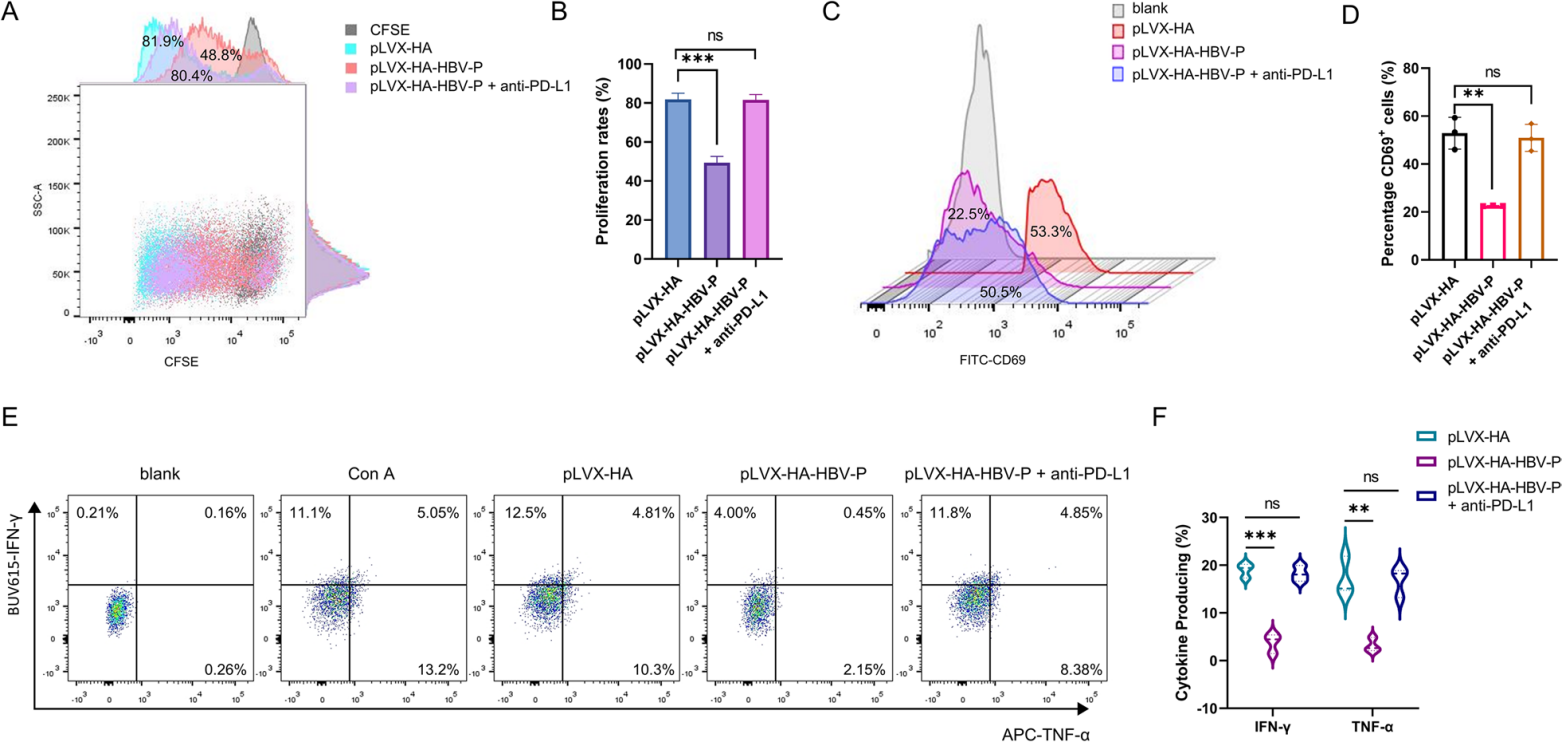
PD-L1 blockade reverses the inhibition of HBV DNA polymerase on the function of Jurkat cells. **A**, **B** PD-L1 blockade reverses the inhibition of HBV DNA polymerase on the proliferation of Jurkat cells. **A** Jurkat cells were directly co-cultured with HBV-DNA-Pol^+^ Huh7 cells for 24 h in the presence of anti-PD-L1(5 μg/ml) or isotype control, then isolated and stained with CFSE, proliferation rates were measured by flow cytometry after 48 h of Con A stimulation. **B** The results were plotted after quantitation. **C**, **D** PD-L1 blockade reverses the inhibition of HBV DNA polymerase on the activation of Jurkat cells. **C** Jurkat cells were directly co-cultured with HBV-DNA-Pol^+^ Huh7 cells for 24 h in the presence of anti-PD-L1(5 μg/ml) or isotype control, then isolated and CD69 expression was determined by flow cytometry after 24 h of Con A stimulation. **D** The quantified results were plotted. **E**, **F** PD-L1 blockade reverses the inhibition of HBV DNA polymerase on the cytokine secretion of Jurkat cells. **E** Jurkat cells were directly co-cultured with HBV-DNA-Pol^+^ Huh7 cells for 24 h in the presence of anti-PD-L1(5 μg/ml) or isotype control, then isolated to measure the production of IFN-γ and TNF-a by flow cytometry 24 h after Con A stimulation. **F** IFN-γ and TNF-a production was summarized. In all statistical comparisons, three independent experiments were performed (mean ± S.D., n = 3, Student’s t-test). **P < 0.01, ***P < 0.001, ns, no significant

To test whether pLVX-HA-HBV-P cells inhibit the function of Jurkat cells via PD-L1, we used PD-L1 antibody to block PD-L1 simultaneously in a previous direct co-culture system and then tested the same indices. The results showed that PD-L1 antibody blockade reversed the inhibitory effects of pLVX-HA-HBV-P cells on the proliferation (Fig. 5A, B and Additional file 1: Fig. S4A), activation (Fig. 5C, D and Additional file 1: Fig. S4B), and cytokine secretion (Fig. 5E, F and Additional file 1: Fig. S4C, D) functions of Jurkat cells. Together, these data suggest that the inhibitory effect of pLVX-HA-HBV-P cells on Jurkat cells is mediated by PD-L1, and that PD-L1 antibody blockade promotes the restoration of immune function in the co-culture system. Given that the regulation of PD-L1 by HBV-DNA-Pol depends on its interaction with PARP1, the deletion of the corresponding domain is equivalent to blocking PD-L1, and the inhibition of immune cell function by HBV-DNA-Pol should be alleviated at this time. In order to validate this point, we also carried out immune function-related experiments after the deletion of the corresponding domain, and the results (Additional file 1: Fig. S5A–F) showed that the inhibition of immune cell function by HBV-DNA-Pol was alleviated after the deletion of the corresponding domain, which confirms the results of our previous experiments.

### Impaired T-cell function in HBV-DNA-Pol transgenic mice

**Fig. 6:**
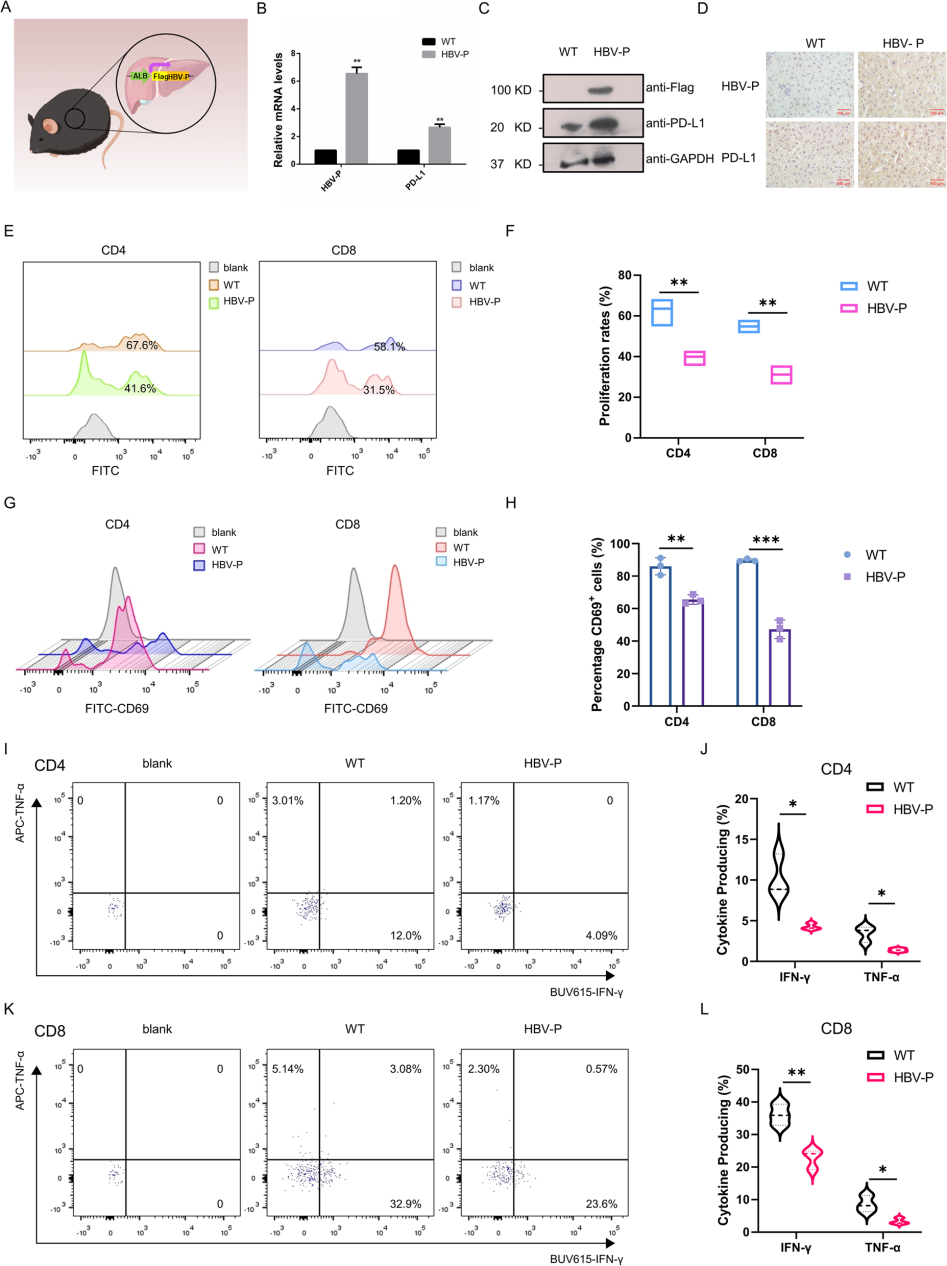
Histopathological and immunological examination of HBV-DNA-Pol transgenic mice. **A** Pattern diagram of HBV-DNA-Pol transgenic mice drawn by Figdraw. Specific expression of HBV-DNA-Pol in liver tissue using albumin as a promoter. **B** Elevated PD-L1 mRNA levels in liver tissues of HBV-DNA-Pol transgenic mice. RNA extracts of liver tissues from HBV-DNA-Pol transgenic mice or wild-type mice were harvested and analyzed for PD-L1 mRNA levels. GAPDH mRNAs were used as a control. In all statistical comparisons, three independent experiments were performed (mean ± S.D., n = 3, Student’s t-test). **P < 0.01. **C**, **D** Increased PD-L1 protein levels in liver tissues of HBV-DNA-Pol transgenic mice. **C** Extracts of liver tissues from HBV-DNA-Pol transgenic mice or wild-type mice were collected and subjected to Western blotting with anti-Flag, anti-PD-L1 and anti-GAPDH antibodies. **D** Liver tissues from HBV-DNA-Pol transgenic mice or wild-type mice were subjected to IHC staining with antibody against Flag and PD-L1. Representative images are displayed. Scale bar, 100 μm. **E**, **F** Impaired proliferation of CD4^+^ and CD8^+^ T cells in the spleen of HBV-DNA-Pol transgenic mice. **E** Immune cells isolated from the spleens of HBV-DNA-Pol transgenic mice or wild-type mice were stimulated for 48 h, proliferation rates of CD4^+^ and CD8^+^ T cells were measured by flow cytometry. **F** The results were plotted after quantitation. **G**, **H** Impaired activation of CD4^+^ and CD8^+^ T cells in the spleen of HBV-DNA-Pol transgenic mice. **G** Immune cells isolated from the spleens of HBV-DNA-Pol transgenic mice or wild-type mice were stimulated for 24 h, CD69 expression of CD4^+^ and CD8^+^ T cells were measured by flow cytometry. **H** The quantified results were plotted. **I**, **J** Impaired cytokine secretion of CD4^+^ T cells in the spleen of HBV-DNA-Pol transgenic mice. **I** Immune cells isolated from the spleens of HBV-DNA-Pol transgenic mice or wild-type mice were stimulated for 24 h, IFN-γ and TNF-a production of CD4^+^ T cells were measured by flow cytometry. **J** IFN-γ and TNF-a production was summarized. **K**, **L** Impaired cytokine secretion of CD8^+^ T cells in the spleen of HBV-DNA-Pol transgenic mice. **K** Immune cells isolated from the spleens of HBV-DNA-Pol transgenic mice or wild-type mice were stimulated for 24 h, IFN-γ and TNF-a production of CD8^+^ T cells were measured by flow cytometry. **L** IFN-γ and TNF-a production was summarized. In all statistical comparisons, three independent experiments were performed (mean ± S.D., n = 3, Student’s t-test). *P < 0.05, **P < 0.01, ***P < 0.001

To clarify the role of HBV-DNA-Pol in T cell dysfunction, we established transgenic mice with liver-specific expression of HBV-DNA-Pol. Figure 6A shows the pattern of transgenic mice. DEN is a chemical carcinogen that has been reported in the literature to be used in combination with carbon tetrachloride (CCl4) to induce HCC [26]. Therefore, we treated HBV-DNA-Pol transgenic mice and sibling control mice with DEN and CCl4 to induce hepatocarcinogenesis. The successfully induced transgenic mice and wild-type mice were then selected to detect PD-L1 mRNA and protein levels in liver tissues by qRT-PCR and Western blotting assays. We found that the mRNA (Fig. 6B) and protein (Fig. 6C) levels of PD-L1 were elevated in the liver tissues of HBV-DNA-Pol transgenic mice, which was consistent with our cellular experiments. In addition, immunohistochemical (IHC) analysis also showed higher levels of PD-L1 expression in the liver tissues of HBV-DNA-Pol transgenic mice (Fig. 6D). To clarify the functions of T cells in HBV-DNA-Pol transgenic mice and wild-type mice under HCC conditions, we isolated immune cells from the spleens of both successfully induced mice and then detected the related functions of CD4^+^ and CD8^+^ T cells by flow assay. The results showed that the proliferation (Fig. 6E, F), activation (Fig. 6G, H), and cytokine secretion (Fig. 6I–L) of CD4^+^ and CD8^+^ T cells were inhibited in the spleens of transgenic mice, which was also consistent with the in vitro experiments. Overall, these in vivo observations not only validate our previous findings but also further suggest a role for HBV-DNA-Pol in the immune escape of HCC cells.

## Discussion

**Fig. 7:**
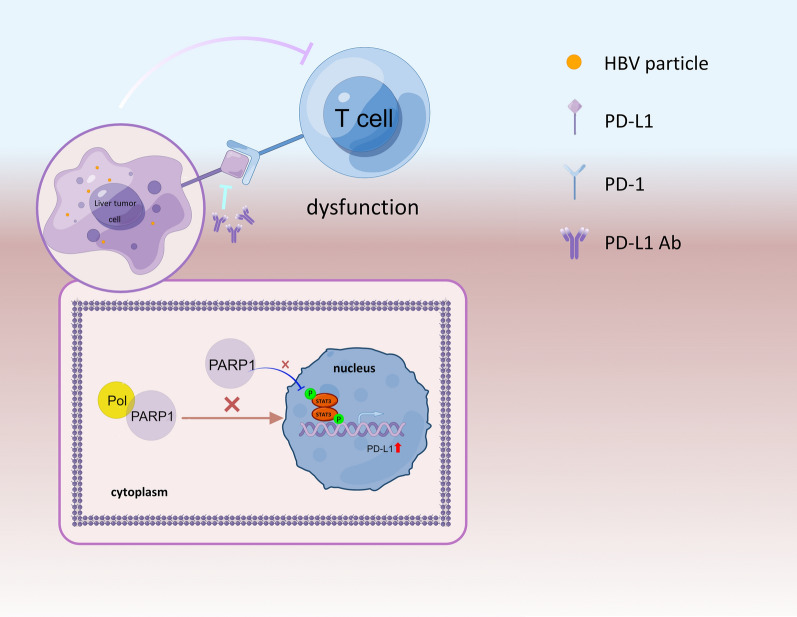
A pattern of immune escape of HBV-associated HCC tumor cells drawn by Figdraw. In HBV-associated HCC, HBV DNA polymerase inhibits the nuclear translocation of PARP1 by interacting with PARP1, thereby upregulating PD-L1 expression in tumor cells at the transcriptional level. PD-L1 on the surface of tumor cells then binds to PD-1 on the surface of T cells, which in turn inhibits T cell function and promotes HCC development

In the current study, we determined that PD-L1, a critical immune checkpoint protein, is upregulated by HBV-DNA-Pol. Specifically, HBV-DNA-Pol regulates PD-L1 transcription by repressing PARP1 nuclear translocation, which results in excessive PD-L1 expression in malignant hepatocytes, leading to PD-L1/PD-1 axis-mediated tumor immune evasion, and ultimately, HCC development (Fig. 7). Currently, very few examples of definitive biological roles for the HBV encodes proteins exist in T-cell exhaustion of HBV-related HCC. Our results provide a clear example of how HBV-DNA-Pol affects the molecular and biochemical functions of PDL-1 as well as the biological outcomes that it controls. More broadly, our results connected HBV-DNA-Pol with the regulation of cancer immune suppression.

HCC is a highly heterogeneous disease with varying immune microenvironments between tumor and adjacent tissues [27]. Immune cells in the tumor microenvironment are mostly functionally suppressed, especially T cells, which often show functional exhaustion, allowing tumor cells to evade the surveillance of the immune system and tumor progression. Studies have shown that T cell exhaustion is often associated with persistent infection and inefficient tumor control [28]. Based on this, we examined the effects of HBV-DNA-Pol on the proliferation, activation, and cytokine secretion of Jurkat cells and found that HBV-DNA-Pol suppressed the immune function of Jurkat cells, confirming the findings of previous studies. Similarly, analysis of an HBV-DNA-Pol transgenic mouse model showed that HBV-DNA-Pol has the potential to cause T cells to enter a state of exhaustion. Together, these results suggest that HBV-DNA-Pol may play an important role in T cell exhaustion, which leads to the immune escape of HBV and HCC tumor cells. Furthermore, aberrant expression of immune checkpoint molecules on the surface of tumor cells is a key feature of T-cell exhaustion; therefore, we analyzed the expression of the relevant immune checkpoint molecules in HBV-DNA-Pol-overexpressing HCC cells and screened for the differentially expressed molecule PD-L1.

PD-L1 is a key mediator of the interactions between T lymphocytes and tumor cells in the tumor microenvironment [29]. Anti-PD-L1 and anti-PD-1 antibodies have been used to treat cancers. However, only a fraction of patients responded to these treatments [30]. Therefore, anti-PD-L1/PD-1 therapy can be improved by understanding PD-L1 regulation and may also identify better biomarkers as well as treatment targets for cancer therapy. In the past decades, the expression of PD-L1 could be artificially stimulated by different cytokines through the corresponding signaling pathways. The activation of downstream transcriptional regulators and nuclear translocation can be enhanced by these signaling pathways, leading to increased PD-L1 transcription [31, 32]. Additionally, intrinsic oncogenic changes can induce PD-L1 expression. For example, the transcription factor AP-1 promotes PD-L1 expression in Hodgkin lymphoma by binding to the AP-1-responsive enhancer of the PD-L1 gene [33]. Our experiments in HCC cells showed that HBV-DNA-Pol increased PD-L1 mRNA and protein expression levels. To further determine whether the upregulation of PD-L1 by HBV-DNA-Pol occurred at the transcriptional or post-transcriptional level, we performed mRNA and protein half-life experiments, which showed that the overexpression of HBV-DNA-Pol did not affect the half-life of PD-L1. These data suggest that upregulation of PD-L1 by HBV-DNA-Pol is due to the promotion of its transcription. Furthermore, our functional experiments showed that HBV-DNA-Pol affected the immunosuppression of T cells to some extent, and this effect was reversed after blocking with the PD-L1 antibody, which is similar to the work of Martinez et al. [34], confirming that HBV-DNA-Pol plays an immunosuppressive role in HCC by upregulating the transcription of PD-L1. However, HBV-DNA-Pol is not a transcription factor; therefore, we aimed to identify endogenous transcriptional regulators of PD-L1 associated with HBV-DNA-Pol in HCCs.

Proteins that bind specifically to HBV-DNA-Pol in Huh7 cells were identified by mass spectrometry, and PARP1 was one of the candidates that intrigued us most. PARP1 is the most characterized member of the PARPs, which uses NAD^+^ as a cofactor to promote PARylation of other proteins, thereby controlling a range of different biological functions [35]. The first clear role of PARP1 is in DNA damage repair [36]. However, subsequent studies have shown that PARP1 has multiple cellular functions and plays a key role in the biological processes of many cancers, including the maintenance of genome integrity, DNA methylation, transcriptional regulation, circadian regulation, chromatin regulation, and histone modification [16, 24, 37–39]. Given its biological importance and potential as a cancer drug target, PARP1 has become a focal point in oncology, and research on it has been intensifying. Some PARP1 inhibitors have been put into the clinic for cancer treatment, including breast, ovarian, and gastric cancers [40]. PARP1 inhibitors for other cancers, such as HCC, are also being investigated in clinical trials. Among these PARP1-related studies, the one that caught our attention the most was a recent study that showed that intrinsic TLR9 activation in hepatocellular carcinoma cells downregulates PARP1 expression, which in turn downregulates STAT3 PARylation, leading to increased STAT3 phosphorylation, resulting in upregulation of PD-L1 and ultimately inducing immune escape [24]. Based on this, we speculated that HBV-DNA-Pol regulation of PD-L1 is achieved through PARP1. Co-IP assays confirmed that HBV-DNA-Pol could bind to PARP1. Furthermore, we found that the RT domain is responsible for the interaction between HBV-DNA-Pol and PARP1 and that the AMD domain of PARP1 interacts with HBV-DNA-Pol. Co-localization of HBV-DNA-Pol and PARP1 in the cytoplasm was further confirmed by immunofluorescence analysis. We initially expected that HBV-DNA-Pol would downregulate PARP1 expression. However, our data showed that HBV-DNA-Pol did not alter PARP1 expression levels, but HBV-DNA-Pol inhibited PARP1 nuclear translocation by interaction, which resulted in upregulation of PD-L1 expression, as the up-regulation of PD-L1 by HBV-DNA-Pol was restored when the RT domain interacting with PARP1 in HBV-DNA-Pol was deleted.

Taken together, according to our study, HBV DNA polymerase inhibits the nuclear translocation of PARP1 by interacting with PARP1, thereby upregulating the expression of PD-L1 in tumor cells at the transcriptional level. PD-L1 on the surface of tumor cells then binds to PD-1 on the surface of T cells, thereby inhibiting T cell function and promoting the development of HCC. Although there are still imperfections in our study, we have furthered our understanding of the biological function of HBV-DNA-Pol and discovered a novel mechanism for its regulation of PD-L1, which also brings new ideas to the clinic to improve therapeutic strategies for hepatocellular carcinoma.

## Conclusion

In HBV-associated HCC, HBV DNA polymerase inhibits the nuclear translocation of PARP1 by interacting with PARP1, thereby upregulating the expression of PD-L1 in tumor cells at the transcriptional level. PD-L1 on the surface of tumor cells then binds to PD-1 on the surface of T cells, thereby inhibiting T cell function and promoting HCC development.

### Supplementary Information

**Additional file 1: Figure S1.** HBV-DNA-Pol^+^ Huh7 cells inhibited the function of Jurkat cells. **A.** Validation of stable transfer cell lines. Protein or RNA extracts from the HBV-DNA-Pol^+^ Huh7 cells or control cells were harvested and HBV-P expression was analyzed by Western blotting (upper) or qRT-PCR (lower) assays. GAPDH protein or mRNAs were used as a control. In all statistical comparisons, three independent experiments were performed (mean ± S.D., n = 3, Student’s t-test). ***, P < 0.001. **B.** HBV DNA polymerase inhibits the proliferation of Jurkat cells after direct co-culture. Jurkat cells were directly co-cultured with HBV-DNA-Pol^+^ Huh7 cells or control cells for 24 h, then isolated and cell viability was detected by MTT assays at 24 h and 48 h after Con A stimulation, respectively. **C.** HBV DNA polymerase inhibits the activation of Jurkat cells after direct co-culture. Jurkat cells were directly co-cultured with HBV-DNA-Pol^+^ Huh7 cells or control cells for 24 h, then isolated and RNA extracts were harvested after 24 h of Con A stimulation and CD69 levels were analyzed by qRT-PCR.** D** and** E**. HBV DNA polymerase inhibits the cytokine secretion of Jurkat cells after direct co-culture. Jurkat cells were directly co-cultured with HBV-DNA-Pol^+^ Huh7 cells or control cells for 24 h, then isolated to measure the production of IFN-γ **(D)** and TNF-a **(E)** by ELSA 24 h after Con A stimulation. **F.** HBV DNA polymerase does not impair the proliferation of Jurkat cells after indirect co-culture. Jurkat cells were indirectly co-cultured with HBV-DNA-Pol^+^ Huh7 cells or control cells for 24 h, then isolated and cell viability was detected by MTT assays at 24 h and 48 h after Con A stimulation, respectively. **G.** HBV DNA polymerase do not impair the activation of Jurkat cells after indirect co-culture. Jurkat cells were indirectly co-cultured with HBV-DNA-Pol^+^ Huh7 cells or control cells for 24 h, then isolated and RNA extracts were harvested after 24 h of Con A stimulation and CD69 levels were analyzed by qRT-PCR.** H** and** I**. HBV DNA polymerase do not impair the cytokine secretion of Jurkat cells after indirect co-culture. Jurkat cells were indirectly co-cultured with HBV-DNA-Pol^+^ Huh7 cells or control cells for 24 h, then isolated to measure the production of IFN-γ **(H)** and TNF-a **(I)** by ELSA 24 h after Con A stimulation. In all statistical comparisons, three independent experiments were performed (mean ± S.D., n = 3, Student’s t-test). ***, P < 0.001, ns, no significant. **Figure S2.** HBV-DNA-Pol upregulates PD-L1 expression. **A.** PD-L1 protein levels in different hepatocellular carcinoma cell lines. Extracts from different hepatocellular carcinoma cell lines were collected and subjected to Western blotting with anti-PD-L1 and anti-GAPDH antibodies. **B.** HBV-DNA-Pol increases PD-L1 protein levels. HepG2.2.15 cells were transfected with the indicated plasmids. Extracts were collected at 48 h post-transfection and PD-L1 expression was analyzed by Western blotting. **C.** HBV-DNA-Pol increases PD-L1 protein levels in a somewhat dose-dependent manner. Huh7 cells were transfected with the indicated plasmids. Extracts were collected at 48 h post-transfection and PD-L1 expression was analyzed by Western blotting. **Figure S3.** HBV-DNA-Pol upregulates PD-L1 expression via PARP1 and STAT3. **A.** HBV-DNA-Pol upregulates PD-L1 expression via PARP1. pLVX-HA-HBV-P cells were transfected with the indicated plasmids. Extracts were collected at 48 h post-transfection and PD-L1 expression was analyzed by Western blotting and qRT-PCR assays. **B.** HBV-DNA-Pol upregulates PD-L1 expression via STAT3. pLVX-HA-HBV-P cells were transfected with the indicated plasmids. Extracts were collected at 48 h post-transfection and PD-L1 expression was analyzed by Western blotting and qRT-PCR assays. **Figure S4.** PD-L1 blockade reverses the inhibition of HBV DNA polymerase on the function of Jurkat cells. **A.** PD-L1 blockade reverses the inhibition of HBV DNA polymerase on the proliferation of Jurkat cells. Jurkat cells were directly co-cultured with HBV-DNA-Pol^+^ Huh7 cells for 24 h in the presence of anti-PD-L1 (5 μg/ml) or isotype control, then isolated and stained with CFSE, proliferation rates were measured by flow cytometry after 48 h of Con A stimulation. **B.** PD-L1 blockade reverses the inhibition of HBV DNA polymerase on the activation of Jurkat cells. Jurkat cells were directly co-cultured with HBV-DNA-Pol^+^ Huh7 cells for 24 h in the presence of anti-PD-L1 (5 μg/ml) or isotype control, then isolated and CD69 expression was determined by flow cytometry after 24 h of Con A stimulation.** C** and** D**. PD-L1 blockade reverses the inhibition of HBV DNA polymerase on the cytokine secretion of Jurkat cells. Jurkat cells were directly co-cultured with HBV-DNA-Pol^+^ Huh7 cells for 24 h in the presence of anti-PD-L1 (5 μg/ml) or isotype control, then isolated to measure the production of IFN-γ **(C)** and TNF-a **(D)** by flow cytometry 24 h after Con A stimulation. In all statistical comparisons, three independent experiments were performed (mean ± S.D., n = 3, Student’s t-test). **, P < 0.01, ***, P < 0.001, ns, no significant. **Figure S5.** Inhibition of immune cell function by HBV-DNA-Pol is dependent on its interaction with PARP1.** A** and** B**. RT domain deletion reverses the inhibition of HBV DNA polymerase on the proliferation of Jurkat cells. **(A)** Huh7 cells were transfected with the indicated plasmids and directly co-cultured with Jurkat cells for 24 h, then isolated and stained with CFSE, proliferation rates were measured by flow cytometry after 48 h of Con A stimulation. **(B)** The results were plotted after quantitation.** C** and** D**. RT domain deletion reverses the inhibition of HBV DNA polymerase on the activation of Jurkat cells. **(C)** Huh7 cells were transfected with the indicated plasmids and directly co-cultured with Jurkat cells for 24 h, then isolated and CD69 expression was determined by flow cytometry after 24 h of Con A stimulation. **(D)** The quantified results were plotted.** E** and** F**. RT domain deletion reverses the inhibition of HBV DNA polymerase on the cytokine secretion of Jurkat cells. **(E)** Huh7 cells were transfected with the indicated plasmids and directly co-cultured with Jurkat cells for 24 h, then isolated to measure the production of IFN-γ and TNF-a by flow cytometry 24 h after Con A stimulation. **(F)** IFN-γ and TNF-a production was summarized. In all statistical comparisons, three independent experiments were performed (mean ± S.D., n = 3, Student’s t-test). **, P < 0.01, ***, P < 0.001, ns, no significant.

## Data Availability

All relevant data supporting the findings of this study are available in the article, additional files, or from the corresponding authors upon reasonable request.

## Abbreviations

AFP: Alpha-fetoprotein
BRCT: Breast cancer type 1 susceptibility protein C-terminus
B7H3: B7 Homolog 3
CCl4: Carbon tetrachloride
Con A: Concanavalin A
DEN: *N*-Nitrosodiethylamine
HBV: Hepatitis B Virus
HBV-DNA-Pol: HBV DNA polymerase
HCC: Hepatocellular carcinoma
ICAM1: Intercellular cell adhesion molecule-1
IDO: Indoleamine2,3-dioxygenase
IHC: Immunohistochemical
PARP1: Poly(ADP-ribose) polymerase 1
PD-1: Programmed death-1
PD-L1: Programmed cell death-ligand 1
RH: RNase H-like
RT: Reverse transcriptase
STAT: Signal transducer and activator of transcription
TLR9: Toll-like receptor 9
TP: Terminal protein
