# Supplementary material for: HBV DNA polymerase upregulates the transcription of PD-L1 and suppresses T cell activity in hepatocellular carcinoma
Source: J Transl Med. 2024 Mar 12;22:272. doi: 10.1186/s12967-024-05069-y (PMC10936085; doi:10.1186/s12967-024-05069-y)
Supplement: Supplementary file 1 — Additional file 1: Figure S1. HBV-DNA-Pol+ Huh7 cells inhibited the function of Jurkat cells. A. Validation of stable transfer cell lines. Protein or RNA extracts from the HBV-DNA-Pol+ Huh7 cells or control cells were harvested and HBV-P expression was analyzed by Western blotting (upper) or qRT-PCR (lower) assays. GAPDH protein or mRNAs were used as a control. In all statistical comparisons, three independent experiments were performed (mean ± S.D., n = 3, Student’s t-test). ***, P < 0.001. B. HBV DNA polymerase inhibits the proliferation of Jurkat cells after direct co-culture. Jurkat cells were directly co-cultured with HBV-DNA-Pol+ Huh7 cells or control cells for 24 h, then isolated and cell viability was detected by MTT assays at 24 h and 48 h after Con A stimulation, respectively. C. HBV DNA polymerase inhibits the activation of Jurkat cells after direct co-culture. Jurkat cells were directly co-cultured with HBV-DNA-Pol+ Huh7 cells or control cells for 24 h, then isolated and RNA extracts were harvested after 24 h of Con A stimulation and CD69 levels were analyzed by qRT-PCR. D and E. HBV DNA polymerase inhibits the cytokine secretion of Jurkat cells after direct co-culture. Jurkat cells were directly co-cultured with HBV-DNA-Pol+ Huh7 cells or control cells for 24 h, then isolated to measure the production of IFN-γ (D) and TNF-a (E) by ELSA 24 h after Con A stimulation. F. HBV DNA polymerase does not impair the proliferation of Jurkat cells after indirect co-culture. Jurkat cells were indirectly co-cultured with HBV-DNA-Pol+ Huh7 cells or control cells for 24 h, then isolated and cell viability was detected by MTT assays at 24 h and 48 h after Con A stimulation, respectively. G. HBV DNA polymerase do not impair the activation of Jurkat cells after indirect co-culture. Jurkat cells were indirectly co-cultured with HBV-DNA-Pol+ Huh7 cells or control cells for 24 h, then isolated and RNA extracts were harvested after 24 h of Con A stimulation and CD69 levels we [file 12967_2024_5069_MOESM1_ESM.docx]

**Additional file**

**
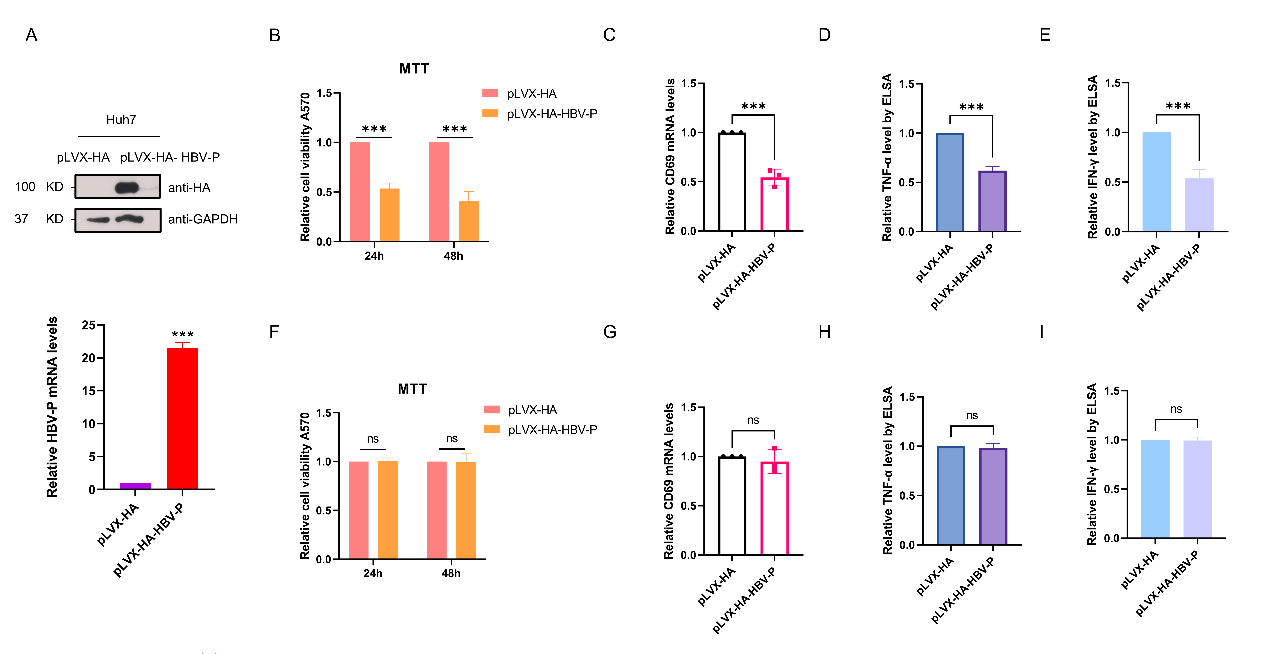
**

**Figure S1. HBV-DNA-Pol^+^ Huh7 cells inhibited the function of Jurkat cells**

**A.** Validation of stable transfer cell lines. Protein or RNA extracts from the HBV-DNA-Pol^+^ Huh7 cells or control cells were harvested and HBV-P expression was analyzed by Western blotting (upper) or qRT-PCR (lower) assays. GAPDH protein or mRNAs were used as a control. In all statistical comparisons, three independent experiments were performed (mean ± S.D., n = 3, Student’s t-test). ***, P < 0.001. **B.** HBV DNA polymerase inhibits the proliferation of Jurkat cells after direct co-culture. Jurkat cells were directly co-cultured with HBV-DNA-Pol^+^ Huh7 cells or control cells for 24 h, then isolated and cell viability was detected by MTT assays at 24 h and 48 h after Con A stimulation, respectively. **C.** HBV DNA polymerase inhibits the activation of Jurkat cells after direct co-culture. Jurkat cells were directly co-cultured with HBV-DNA-Pol^+^ Huh7 cells or control cells for 24 h, then isolated and RNA extracts were harvested after 24 hours of Con A stimulation and CD69 levels were analyzed by qRT-PCR. **D and E.** HBV DNA polymerase inhibits the cytokine secretion of Jurkat cells after direct co-culture. Jurkat cells were directly co-cultured with HBV-DNA-Pol^+^ Huh7 cells or control cells for 24 h, then isolated to measure the production of IFN-γ **(D)** and TNF-a **(E)** by ELSA 24 hours after Con A stimulation. **F.** HBV DNA polymerase does not impair the proliferation of Jurkat cells after indirect co-culture. Jurkat cells were indirectly co-cultured with HBV-DNA-Pol^+^ Huh7 cells or control cells for 24 h, then isolated and cell viability was detected by MTT assays at 24 h and 48 h after Con A stimulation, respectively. **G.** HBV DNA polymerase do not impair the activation of Jurkat cells after indirect co-culture. Jurkat cells were indirectly co-cultured with HBV-DNA-Pol^+^ Huh7 cells or control cells for 24 h, then isolated and RNA extracts were harvested after 24 hours of Con A stimulation and CD69 levels were analyzed by qRT-PCR. **H and I.** HBV DNA polymerase do not impair the cytokine secretion of Jurkat cells after indirect co-culture. Jurkat cells were indirectly co-cultured with HBV-DNA-Pol^+^ Huh7 cells or control cells for 24 h, then isolated to measure the production of IFN-γ **(H)** and TNF-a **(I)** by ELSA 24 hours after Con A stimulation. In all statistical comparisons, three independent experiments were performed (mean ± S.D., n = 3, Student’s t-test). ***, P < 0.001, ns, no significant.


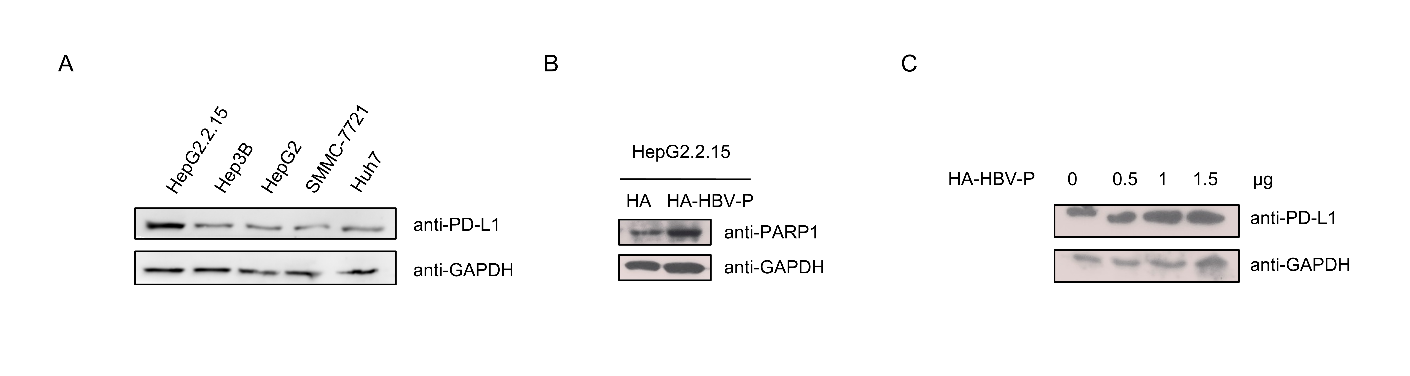


**Figure S2. HBV-DNA-Pol upregulates PD-L1 expression**

**A.** PD-L1 protein levels in different hepatocellular carcinoma cell lines. Extracts from different hepatocellular carcinoma cell lines were collected and subjected to Western blotting with anti-PD-L1 and anti-GAPDH antibodies. **B.** HBV-DNA-Pol increases PD-L1 protein levels. HepG2.2.15 cells were transfected with the indicated plasmids. Extracts were collected at 48 h post-transfection and PD-L1 expression was analyzed by Western blotting. **C.** HBV-DNA-Pol increases PD-L1 protein levels in a somewhat dose-dependent manner. Huh7 cells were transfected with the indicated plasmids. Extracts were collected at 48 h post-transfection and PD-L1 expression was analyzed by Western blotting.

**
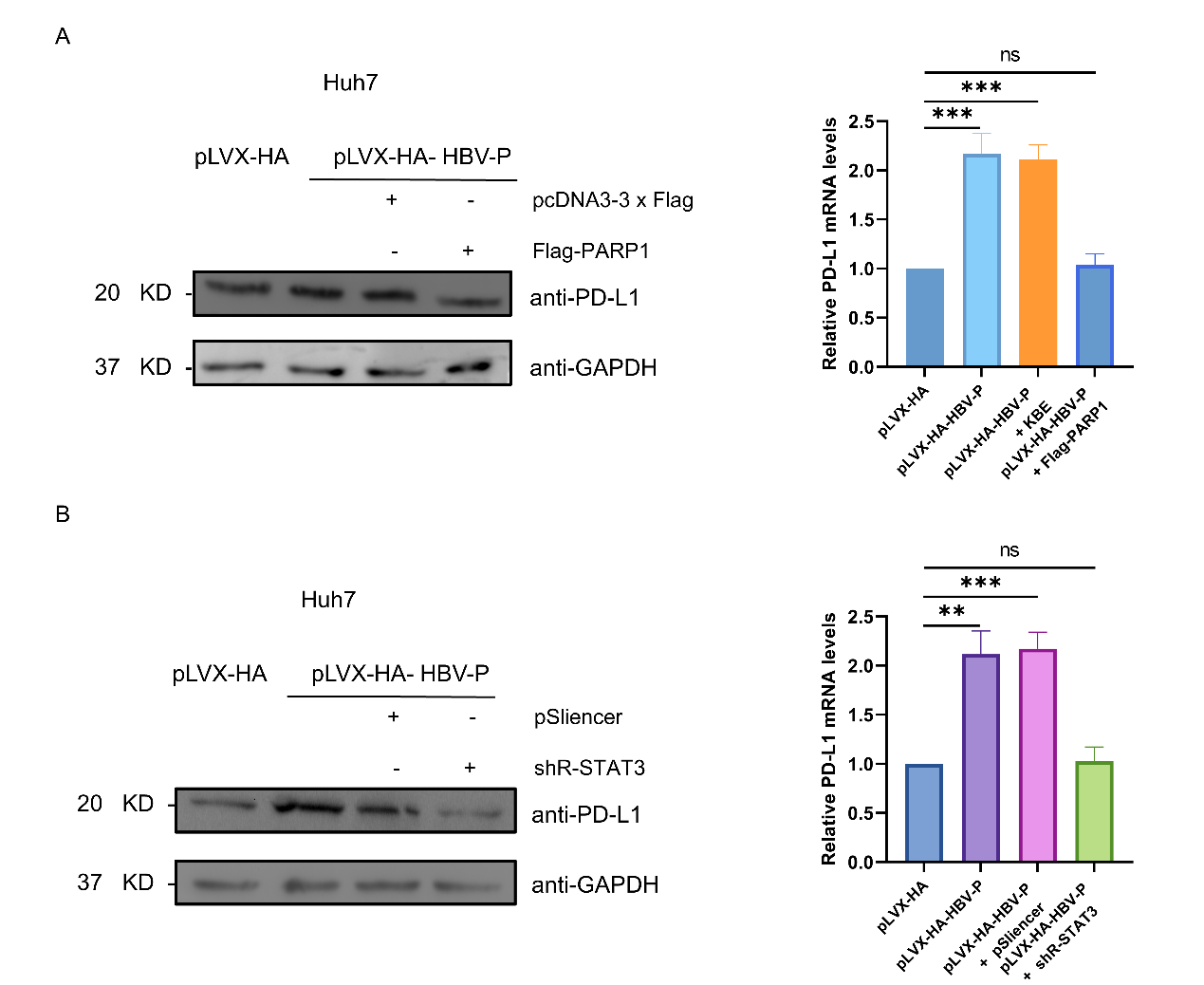
**

**Figure S3. HBV-DNA-Pol upregulates PD-L1 expression via PARP1 and STAT3**

**A.** HBV-DNA-Pol upregulates PD-L1 expression via PARP1. pLVX-HA-HBV-P cells were transfected with the indicated plasmids. Extracts were collected at 48 h post-transfection and PD-L1 expression was analyzed by Western blotting and qRT-PCR assays. **B.** HBV-DNA-Pol upregulates PD-L1 expression via STAT3. pLVX-HA-HBV-P cells were transfected with the indicated plasmids. Extracts were collected at 48 h post-transfection and PD-L1 expression was analyzed by Western blotting and qRT-PCR assays.


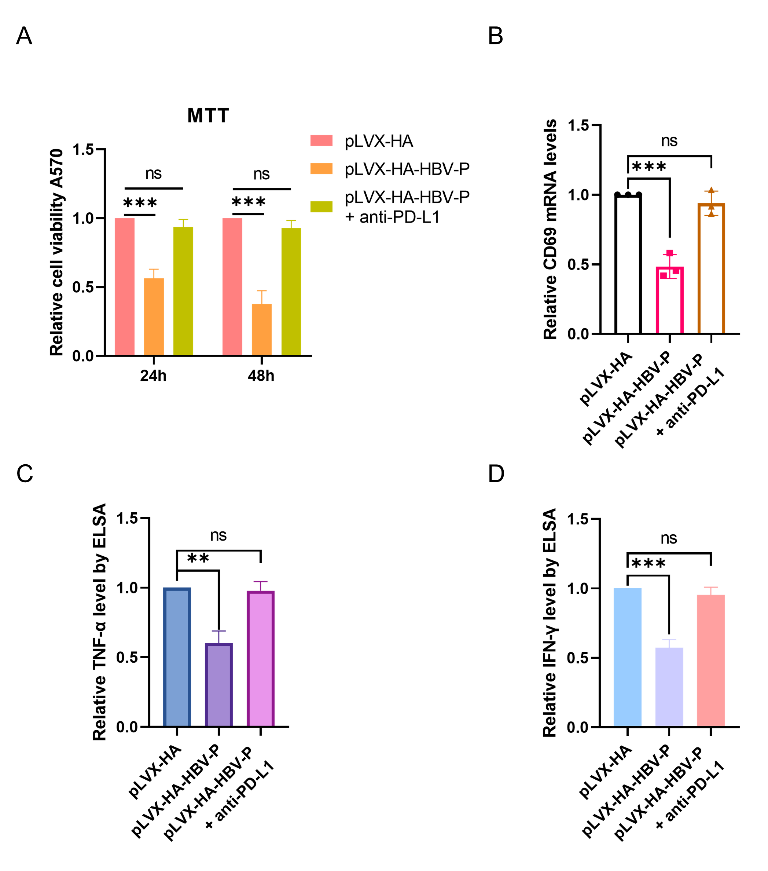


**Figure S4. PD-L1 blockade reverses the inhibition of HBV DNA polymerase on the function of Jurkat cells**

**A.** PD-L1 blockade reverses the inhibition of HBV DNA polymerase on the proliferation of Jurkat cells. Jurkat cells were directly co-cultured with HBV-DNA-Pol^+^ Huh7 cells for 24 h in the presence of anti-PD-L1 （5 μg/ml） or isotype control, then isolated and stained with CFSE, proliferation rates were measured by flow cytometry after 48 hours of Con A stimulation. **B.** PD-L1 blockade reverses the inhibition of HBV DNA polymerase on the activation of Jurkat cells. Jurkat cells were directly co-cultured with HBV-DNA-Pol^+^ Huh7 cells for 24 h in the presence of anti-PD-L1 （5 μg/ml） or isotype control , then isolated and CD69 expression was determined by flow cytometry after 24 hours of Con A stimulation. **C and D.** PD-L1 blockade reverses the inhibition of HBV DNA polymerase on the cytokine secretion of Jurkat cells. Jurkat cells were directly co-cultured with HBV-DNA-Pol^+^ Huh7 cells for 24 h in the presence of anti-PD-L1 （5 μg/ml） or isotype control, then isolated to measure the production of IFN-γ **(C)** and TNF-a **(D)** by flow cytometry 24 hours after Con A stimulation. In all statistical comparisons, three independent experiments were performed (mean ± S.D., n = 3, Student’s t-test). **, P < 0.01, ***, P < 0.001, ns, no significant.


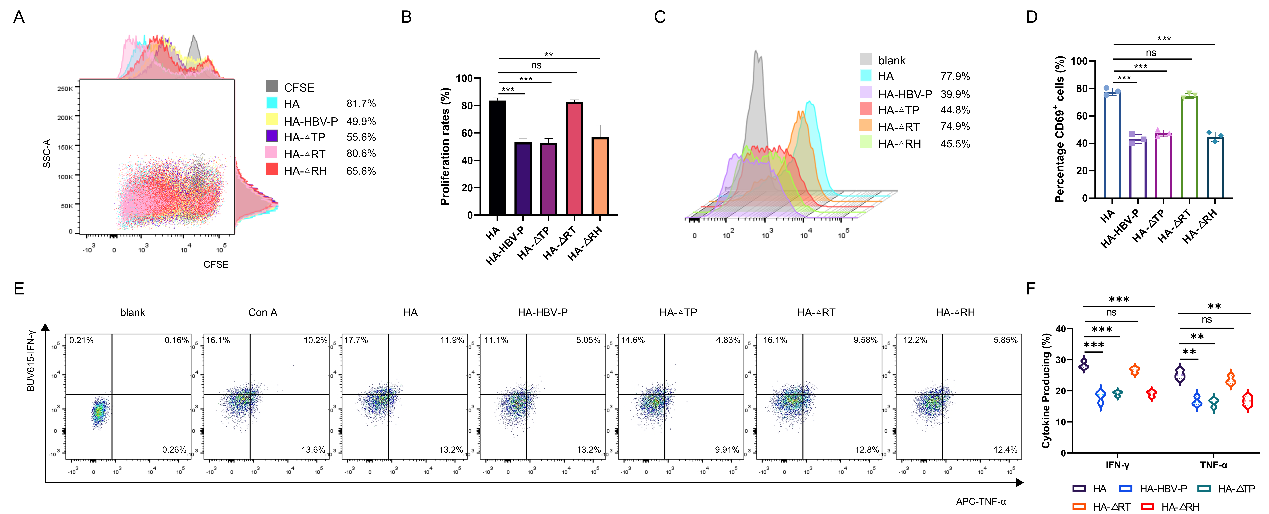


**Figure S5. Inhibition of immune cell function by HBV-DNA-Pol is dependent on its interaction with PARP1**

**A and B.** RT domain deletion reverses the inhibition of HBV DNA polymerase on the proliferation of Jurkat cells. **(A)** Huh7 cells were transfected with the indicated plasmids and directly co-cultured with Jurkat cells for 24 h, then isolated and stained with CFSE, proliferation rates were measured by flow cytometry after 48 hours of Con A stimulation. **(B)** The results were plotted after quantitation. **C and D.** RT domain deletion reverses the inhibition of HBV DNA polymerase on the activation of Jurkat cells. **(C)** Huh7 cells were transfected with the indicated plasmids and directly co-cultured with Jurkat cells for 24 h, then isolated and CD69 expression was determined by flow cytometry after 24 hours of Con A stimulation. **(D)** The quantified results were plotted. **E and F.** RT domain deletion reverses the inhibition of HBV DNA polymerase on the cytokine secretion of Jurkat cells. **(E)** Huh7 cells were transfected with the indicated plasmids and directly co-cultured with Jurkat cells for 24 h, then isolated to measure the production of IFN-γ and TNF-a by flow cytometry 24 hours after Con A stimulation. **(F)** IFN-γ and TNF-a production was summarized. In all statistical comparisons, three independent experiments were performed (mean ± S.D., n = 3, Student’s t-test). **, P < 0.01, ***, P < 0.001, ns, no significant.
